# Use of immunohistochemistry in the differential diagnosis of cutaneous round cell tumours in dogs

**DOI:** 10.1186/1753-6561-7-S2-P50

**Published:** 2013-04-04

**Authors:** Marina R Araújo, Ingred S Preis, Gleidice E Lavale, Geovanni D Cassali, Roselene Ecco

**Affiliations:** 1 Department of General Pathology, Biological Science Institute, Federal University of Minas Gerais, Belo Horizonte, MG, Brazil; 2 Department of Veterinary Pathology, Veterinary School, Federal University of Minas Gerais, Belo Horizonte, MG, Brazil; 3 Veterinary Hospital, Veterinary School, Federal University of Minas Gerais, Belo Horizonte, MG, Brazil

## Background

Cutaneous round cell tumours may have a similar morphological appearance, and a diagnosis based on routine histopathology only is often challenging. Immunohistochemistry has been proven to be one of the most important ancillary techniques in the characterization of neoplastic diseases in veterinary medicine.

## Materials and methods

This work describes an antibody panel (CD117, CD3, CD79a, CD45, cytokeratin, vimentin and E-cadherin) for immunohistochemistry analyses of formalin-fixed, paraffin-embedded sections of canine cutaneous round cell tumours. Neoplastic tumours were diagnosed by histology and histochemical staining and included 89 mast cell tumours, 31 cutaneous histiocytomas, 21 cutaneous lymphomas, three plasma cell tumours, and seven unclassified round cell tumours.

## Results

The histologic diagnosis was modified in 43.5% of the total 147 neoplasms. The staining for CD45 and E-cadherin were variable, and therefore, the final diagnoses of cutaneous histiocytoma was made based on histology in association with negative results for CD3, CD79a, CD117 and cytokeratin. The cellular origin of unclassified round cell tumours was defined in all cases. Cutaneous B-cell lymphoma and plasma cell tumours were CD79a-positive and could be distinguished from each other by the morphological characteristics. Mast cell tumours and T cell lymphoma were CD117and CD3 positive, respectively. The positive staining for vimentin and the negative staining for CD3, CD79a, CD117 and cytokeratin favoured the diagnosis of transmissible venereal tumours.

## Conclusions

The final diagnosis of cutaneous round cell tumours should be based on the interpretation of immunohistochemical results together with the cellular morphology observed by histology. Therefore, more studies to optimize the specific markers in formalin-fixed, paraffin-embedded tissues (especially for histiocytes) are required for definitive diagnosis of round cell tumours in dogs.

## Financial support

PRPq-UFMG, CNPq, FAPEMIG and CAPES.

